# Environmental health perception by Brussels inhabitants: comparison between a top-down raising awareness and citizen science

**DOI:** 10.1093/eurpub/ckac129.080

**Published:** 2022-10-25

**Authors:** P Incourt, L Herbrich, C Bouland

**Affiliations:** School of Public Health, Université Libre de Bruxelles, Brussels, Belgium

## Abstract

The effects of the environment on health are well documented and prove to be a real public health problem. It is therefore essential to raise public awareness of these issues to induce preventive and protective behaviours. We focused on two methods: passive information transmission (top-down approach) and citizen science (bottom-up approach). The study aims to compare both approaches while raising awareness among Brussels citizens. We created two groups: a traditional awareness group, receiving infographics by email, and a citizen science group, carrying out immersive activities with researchers. All enrolled participants filled out a questionnaire before and after. The “top-down” group (n = 137) received 3 infographics. The citizen science participants deep-dived into the environmental health body of knowledge, carried out individual measurements of air quality and noise pollution along a city walk and analysed, together, the results in groups to design actions. The citizen science sessions were finalised by a focus group. All sessions enjoyed and developed knowledge and awareness of environmental health. Accompanying citizens in developing knowledge was beneficial and required for environmental health empowerment. It showed the added value of citizen science in raising curiosity, creativity, and capacity building. The participants showed different socioeconomic statuses and demonstrated an appetite for understanding the exposures measured during the walks. Our results integrate several SDGs among those SDG4 and SDG3, since by raising awareness of participants, we enabled them to improve their capacities in becoming actors in their health. The risk of developing health problems related to the environment is higher in lower socio-economic groups, due to a greater vulnerability and the inequitable environment distribution between neighbourhoods. Pro-environmental behaviour fosters reduced exposure for now and future generations.

